# Effect of orange pulp with or without zeolite on productive performance, nitrogen utilization, and antioxidative status of growing rabbits

**DOI:** 10.1007/s11250-024-04157-x

**Published:** 2024-10-03

**Authors:** Wafaa Mostafa Ali Ghoneem, Hassan Awny Fouad Rahmy, Reham Roshdi Ali El-Tanany

**Affiliations:** Animal Production Department, Faculty of Agriculture, Cairo University, Giza, 12613 Egypt

**Keywords:** Orange pulp, Zeolite, Rabbits, N- utilization, Antioxidants

## Abstract

The current study was designed to investigate the effect of dried orange pulp inclusion (OP diet), natural zeolite addition (Z diet), or both (OPZ diet) compared to control (CON diet) on digestibility, growth performance, nitrogen utilization, blood biochemical, antioxidative status, and cecum microbiota of growing rabbits. Seventy-two V-line male rabbits (6 weeks old) were divided into 4 balanced experimental groups. Results showed that administration of dried orange pulp or zeolite especially the OPZ diet significantly improved nutrient digestibility and nutritive values. Rabbits fed the experimental diets (OP, Z, or OPZ) recorded significantly higher values of average daily gain, N-retention, and N-balance compared with those fed the CON diet. Data on blood biochemical, showed non-significant differences in globulin concentrations, and significant decreases in levels of cholesterol, LDL (low-density lipoproteins), triglycerides, and MDA (malondialdehyde) as an antioxidant biomarker with OP, Z, or OPZ diets. Moreover, the incorporation of orange pulp or zeolite in diets significantly decreased the cecal count of *E. coli*, with no significant difference in total bacterial count among the experimental groups. It could be concluded that a combination between dried orange pulp and natural zeolite in the diet can enhance the growth performance, antioxidant and health status of rabbits.

## Introduction

Rabbits are characterized by their ability to utilize agricultural and agro-industrial by-products as major ingredients in their diets (Effiong et al. 2016). The incorporation of these by-products in animal diets has the potential to reduce the environmental burden (De Blas et al. 2018), and also has a positive economic effect by decreasing the feeding cost (Suliman et al. 2019). Orange fruit pulp (OP) is one of the agro-industrial by-products, which is composed of orange seeds, peels, and pulp. Orange pulp acts as a natural source of antioxidants such as flavonoids, ascorbic acid (Hassan et al. 2021), and vitamin E (Shanthi et al. 2019). It can be used in the diets of rabbits as an energy source to partially replace yellow corn, barley, or sorghum due to its high energy content being 3756.14 kcal ME/kg (Effiong et al. 2016). The previous data revealed that dried orange pulp can partially replace corn in the rabbit's diet by up to 60% (Ibrahim et al. 2011). However, Zeweil et al. (2015) concluded that OP can replace barley up to 30% with no adverse effect on the rabbit's performance.

Ammonia (NH_3_) is one of the dietary protein metabolism end products from animals. The excess N volatilization can adversely affect the environmental balance as a result of nitrous oxide formation, which has a negative impact on climate change (Ndegwa et al. 2008). Also, animals and rabbits particularly are sensitive to high NH_3_ concentrations, which increases respiratory diseases and eye infections (Cui et al. 2021). Several studies conducted on different animals confirmed a negative impact of high NH_3_ concentration on respiratory, immune, reproductive, and nervous systems (Zhao et al. 2018).

The inclusion of OP was confirmed to provide diets with higher proportions of complex carbohydrates such as β-glucans, hemicelluloses, cellulose, and other non-starch polysaccharides (NSP) (Mourão et al. 2008; Hon et al. 2009), which shift excretion of nitrogen from urine to feces and reduces ammonia volatilization (Mroz et al. 2000). Ferrer et al. (2021) reported significant decreases in NH_3_ emission and NH_3_ content in Pigsʼ slurry when OP was included in the diets. Another strategy to reduce NH_3_ volatilization is to use ammonia-binding feed additives, such as natural zeolite in diets of animal, which has a high affinity to ammonia ions (Ndegwa et al. 2008). Reduction in ammonia emission was observed when zeolite was added either to the diets of beef cattle (Bozkurt 2006) or poultry (Burmańczuk et al. 2015). Besides the impact of natural zeolite on NH_3_ volatilization, it has the ability to absorb mycotoxins in feed ingredients and eliminates their effect on animal health and performance (Herc et al. 2021; Abdulrahman et al. 2022).

So, this study hypothesized that a combination between orange pulp and natural zeolite in rabbitsʼ diet may improve the antioxidative status, and nitrogen utilization, which may be reflected in higher growth performance and rabbitsʼ health.

## Materials and methods

### Study area

The present study was conducted at the Rabbit Research Unit, Faculty of Agriculture, Cairo University in January 2022. The chemical analyses were done at laboratories of the Animal Production Department and Faculty of Agriculture Research Park (FARP), Faculty of Agriculture, Cairo University and Blood Lab., Animal Production Research Institute, Ministry of Agriculture, Egypt.

### Rabbits, diets, and the experimental design

Seventy-two of V-line male rabbits (6 weeks old) with average initial body weight ranging from 705.56 to 723.33 g, were divided into 4 experimental groups (18 rabbits/group). Each group contained 6 replicates with 3 rabbits each. All rabbits were housed in metal battery cages (30 × 35 × 40 cm) provided with automatic nipples to supply rabbits with fresh water at all times. All animals were kept under the same conditions of hygiene and management. Diets were offered ad libitum.

The experimental groups were randomly assigned to feed the experimental diets. The first group (CON) received a pellet diet as the control group. The second group (OP) received the pellet diet with 19% orange pulp replacement. The third group (Z) received CON + 1% natural zeolite. While the fourth group (OPZ) received OP diet + 1% natural zeolite. Diet formulation and the chemical composition of orange pulp and the experimental diets are presented in Tables 1 and 2. Diets were formulated to be isocaloric (~ 2500 kcal/DE kg diet) and isonitrogenous (~ 17% CP) and to cover the nutrient requirements as described by Lebas (2013) for growing rabbits. Digestible energy for all diets was estimated as described by Cheeke (1987) as follows:

**Table 1:** Feed ingredients of the experimental diets

Ingredients,%	Experimental diets	
	CON & Z	OP & OPZ
Barley	30.00	14.00
Soybean meal (44% CP)	12.50	15.50
Wheat bran	29.00	26.56
Clover hay	22.55	19.50
Orange pulp	0	19.00
DL-Methionine	0.10	0.11
Di-calcium phosphate	1.60	1.42
NaCl	0.60	0.40
Permix*	0.30	0.30
Anti-coccidia and fungi	0.20	0.20
Limestone	0.15	0.01
Molasses	3.00	3.00
Total	100	100

CON: control diet, Z: control diet + 1% zeolite, OP: 19% orange pulp diet, OPZ: 19% orange pulp + 1% zeolite. * each 1 kg supplied the diet with: 12,000 IU (Vit. A), 2000 IU (Vit. D_3_), 10 mg (Vit. E), 2 g (Vit. K_3_), 1000 mg (Vit. B_1_), 5 mg (Vit. B_2_), 1.5 mg (Vit. B_6_), 10 mg (Vit. B_12_), 30 mg (Niacin), 10 mg (Pantothenic acid), 1 mg (Folic acid), 250 mg (Choine), 50 mg (Biotin), 5 mg (Cu), 60 mg (Mn), 50 mg (Zn), 30 mg (Fe), 0.3 mg (I), 0.1 mg (Se), and 0.1 mg (Co). CON: control diet, Z: control diet + 1% zeolite, OP: 19% orange pulp diet, OPZ: 19% orange pulp + 1% zeolite

**Table 2:** Chemical composition of dried orange pulp and the experimental diets (DM basis)

Items	Dried orange pulp	Experimental diets			
		CON	OP	Z	OPZ
DM	90.3	92.43	92.24	92.15	92.27
OM	94.75	90.47	91.55	90.71	90.34
CP	6.64	17.34	17.23	17.32	17.17
CF	11.99	13.19	13.31	13.25	13.33
EE	2.13	2.34	2.41	2.63	2.73
Ash	5.25	9.53	8.45	9.29	9.66
NFE	73.99	57.60	58.60	57.51	57.11
NDF	21.61	32.91	34.50	32.99	34.53
ADF	19.69	16.75	18.91	16.77	18.87
ADL	2.75	5.55	3.60	5.53	3.54
Hemicellulose	1.92	16.16	15.59	16.22	15.66
Cellulose	16.94	11.20	15.31	11.24	15.33
DE (kcal/kg DM)	2556.73	2518.15	2514.24	2516.17	2513.59
Methionine^1^	0.08	0.69	0.72	0.69	0.72
Lysine^2^	0.17	0.82	0.83	0.82	0.83
Calcium^3^	0.78	1.27	1.22	1.27	1.22
Phosphours^4^	0.10	0.79	0.72	0.79	0.72

CON: control diet, Z: control diet + 1% zeolite, OP: 19% orange pulp diet, OPZ: 19% orange pulp + 1% zeolite. Hemicellulose = NDF – ADF, Cellulose = ADF – ADL. ^1–4^ calculated for diets based on ingredients composition

$$\mathrm{DE}\;(\mathrm{kcal}/\mathrm{kg}=4.36-0.049\ast\left[28.924+0.657(\mathrm{CF}\;\%)\right]$$

The fresh orange pulp by-product was purchased from Juhayna Company, October City, Giza Governate. The orange pulp by-product was sun-dried till 10% moisture. While, natural zeolite was obtained from AandO Trading Company, Egypt.

### Growth performance

During the experimental period (8 weeks), recording of live body weight and calculation of rabbitsʼ weight gain were performed weekly. Feed intake was calculated daily per rabbit as the difference between feed introduced and feed residues collected from each cage. Consequently, the feed conversion ratio (FCR) was calculated as feed intake (g)/gain (g).

### Digestion trial and urine collection

Twenty-four male V-line rabbits (6 rabbits from each group) were taken to perform a digestion trial (Pérez et al. 1995). The collection period lasted for 4 days. Feces and urine were collected separately by using a 4 mm mesh for feces collection and a lower polyethylene layer to collect the urine into a 1-L bottle. Urine was kept and collected separately from each rabbit in a plastic container. 100 ml of 50% sulphuric acid (H_2_SO_4_) was added to the container to avoid nitrogen volatilization. Feces were dried at 70° C/24 h. then the grinded samples were kept until chemical analysis.

Digestion coefficients were determined using the AIA method (the acid-insoluble ash) as described by Lee and Hristov (2013) using the following formula:

$$\mathrm{Digestion}\;\mathrm{coefficient}=100-\left[100\times\frac{\%\;indicator\;in\;feed}{\%\;indicator\;in\;feces}\times\frac{\%\;nutrient\;in\;feces}{\%\;nutrient\;in\;feed}\right]$$

The nutritive values of the diets (TDN and DCP) were calculated as recommended by Cheeke et al. (1982). Digestible energy was calculated as described by Schneider and Flatt (1975) as follows:

$$\text{DE }(\text{kcal}/\text{kg})=\text{TDN}*44.3$$

### Chemical analyses

Dry matter (DM), crude protein (CP), crude fiber (CF), ether extract (EE), and ash were determined in samples of orange pulp by-products, diets, and dried feces according to AOAC (2016). Organic matter (OM) = 100 -ash%. Nitrogen-free extract (NFE) = [100 – (CP% + CF% + EE %)]. Fiber fractions; NDF (neutral detergent fiber), ADF (acid detergent fiber), and ADL (acid detergent lignin); were determined by the method of Van Soest et al. (1991). Hemicellulose = NDF-ADF.

### Slaughtering and cecum characteristics

Six rabbits were randomly chosen from each group at the end of the trial. Rabbits fasted for 12 h, then slaughtering was taken place after recording the rabbits' weight individually. Slaughter procedures were carried out according to Blasco and Ouhayoun (1996). The rabbits’ pelt and viscera were removed after complete bleeding. The cecum from all rabbits was removed, then the cecum contents were collected and divided into 2 parts. The first part was used to determine pH using a digital pH meter (Hanna Instruments Inc., USA, HI98103), While, the second part was put in a bottle and stored at -20 ◦C (Hassan et al. 2020) until the determination of cecum microbiota. Then the empty weight and length of the cecum were measured. The total bacterial count was determined according to ISO (2013), while the method of Feng et al. (2017) was used in the determination of *Escherichia coli*.

### Blood biochemical parameters and antioxidative status

During slaughtering, blood samples were collected in clean tubes containing heparin, and centrifuged for 15 min at 3000 rpm to obtain blood plasma. The total plasma protein concentrations were estimated as described by Tietz (2006). The concentrations of albumin were determined as Doumas et al. (1971) recommended. Globulin was calculated as the difference between total protein and albumin concentrations. Lipids profile (total lipids, cholesterol, LDL, HDL, and triglycerides) were measured as recommended by Zöllner and Kirsch (1962); Richmond (1973); Wieland and Seidel (1983), Bachorik et al. (1982) and Young (2000), respectively. The concentrations of alanine aminotransferase (ALT) and aspartate aminotransferase (AST) were estimated according to Reed (2013). Blood T-AOC (total antioxidant capacity) was analyzed as described by Koracevic et al. (2001). Blood MDA (malondialdehyde) was assayed according to Satoh (1978).

### Statistical analysis

The statistical analysis of the obtained results was done by the general linear model procedure of SAS (2009) using the following model:

$$\text{Yij}=\upmu +\text{Ti}+\text{Eij},$$

Where: μ is the overall mean of Yij; Ti is the treatment effect; Eij is the random error of the experiment. The differences among averages were identified at 5% significance level using Duncan’s New Multiple Range Test.

## Results

### Nutrients digestibility and nutritive value

As shown in Table 3, the inclusion of dried orange pulp (OP) in rabbitʼs diets either alone (OP diet) or in combination with natural zeolite (OPZ diet) significantly (P < 0.05) increased digestion coefficients of dry matter (DM), crude fiber (CF) and nitrogen free extract (NFE). There were non-significant differences in the digestion coefficients of organic matter (OM) and crude protein (CP) with the OP diet but they were significantly (P < 0.05) increased with the OPZ diet compared with the CON diet. The OPZ diet recorded the highest significant (P < 0.05) values of nutrient digestibility. Although a diet supplemented with 1% zeolite (Z diet) had no significant effect on the digestibility of DM, OM, and CP, there were significant (P < 0.05) increases in the digestibility of CF and NFE compared with the control. On the other hand, values of ether extract (EE) digestibility were not significantly changed among the different experimental diets. Total digestible nutrients (TDN) and digestible energy (DE), the measurements of relative feed energy values for the animal, were significantly (P < 0.05) higher for diets containing dried OP, zeolite, or both compared with the CON diet. While, digestible crude protein (DCP) was significantly (P < 0.05) increased with the OPZ diet, with no significant differences among OP, Z, and CON diets.

**Table 3:** Effect of dried orange pulp inclusion with or without zeolite on nutrients digestibility and nutritive value

Item	Experimental groups				± SE	*p*-value
	CON	OP	Z	OPZ		
Apparent digestibility, %						
DM	69.98b	71.92^a^	71.72^ab^	73.02^a^	0.88	0.031
OM	71.56b	73.41^ab^	73.32^ab^	74.83^a^	0.93	0.029
CP	74.07c	75.33^b^	74.65^bc^	77.52^a^	0.75	< 0.001
CF	50.36d	54.42^b^	52.43^c^	55.79^a^	1.18	< 0.001
EE	82.70	83.57	80.60	82.39	1.50	0.291
NFE	75.21c	76.73^b^	77.37^ab^	78.11^a^	0.62	0.003
Nutritive values						
TDN, %	67.16^c^	69.72^ab^	69.14^b^	70.42^a^	0.60	0.001
DCP, %	12.84^b^	12.98^b^	12.93^b^	13.31^a^	0.10	0.001
DE, kcal/kg	2975.19^c^	3088.60^ab^	3062.90^b^	3119.61^a^	28.05	0.001

^a^^, b, c, ..^ means in each row with different superscripts vary significantly (P < 0.05). CON: control diet, Z: control diet + 1% zeolite, OP: 19% orange pulp diet, OPZ: 19% orange pulp + 1% zeo

### Growth performance

The effect of inclusion dried OP, zeolite, or a combination of them in rabbit diets on performance is presented in Table 4. Data revealed non-significant differences among the experimental groups either in initial body weight or dry matter intake (DMI). While significant (P < 0.05) increases were observed in both final body weight and average daily gain (ADG) for rabbits fed either OP or OPZ diets compared with those fed CON. While final BW was not significantly affected by feeding rabbits on the Z diet, ADG was significantly (P < 0.05) increased compared with the CON diet. The highest (P < 0.05) values for final BW and ADG (by 6.95 and 10.69%, respectively) were observed with the OPZ diet compared with the control. Although there were no significant differences among the experimental groups on values of feed conversion ratio (FCR), rabbits fed the OPZ diet recorded numerically the best FCR.

**Table 4:** Effect of the experimental diets on the growth performance of rabbits

Item	Experimental groups				± SE	*p*-value
	CON	OP	Z	OPZ		
Initial BW(g/rabbit)	711.11	705.56	723.33	712.22	8.92	0.939
Final BW(g/rabbit)	1994.44^b^	2079.44^a^	2066.67^ab^	2133.06^a^	36.12	0.015
ADG (g/rabbit/day)	22.92^c^	24.53^b^	23.99^b^	25.37^a^	0.51	0.001
DMI(g/rabbit/day)	93.93	95.84	91.86	96.20	1.00	0.235
FCR (g feed/g gain)	4.10	3.91	3.83	3.79	0.18	0.166

^a^^, b, c, ..^ means in each row with different superscripts vary significantly (P < 0.05). CON: control diet, Z: control diet + 1% zeolite, OP: 19% orange pulp diet, OPZ: 19% orange pulp + 1% zeolite. BW: body weight, ADG: average daily gain, DMI: dry matter intake, FCR: feed conversion ratio

### Nitrogen balance

Data in Table 5, showed that neither dried OP incorporation nor zeolite addition in rabbit's diet had significant effects on N-intake, N-excreted either in feces or urine, and urinary-N: fecal-N ratio. However, values of total N-excreted, N-retention, and N-balance were significantly (P < 0.05) higher for experimental groups fed OP, Z, or OPZ diets compared with those fed the CON diet.

**Table 5:** Effect of dried orange pulp inclusion with or without zeolite on N-balance

Item	Experimental groups				± SE	*p*-value
	CON	OP	Z	OPZ		
N-intake (g/rabbit/d)	2.61	2.64	2.61	2.64	0.02	0.240
Fecal-N (g/rabbit/d)	0.51	0.49	0.48	0.46	0.03	0.092
Urinary-N (g/rabbit/d)	0.17	0.14	0.13	0.12	0.03	0.067
Total N excreted (g/rabbit/d)	0.68^a^	0.63^b^	0.61^bc^	0.58^c^	0.02	0.004
N- retention (g/rabbit/d)	1.93^b^	2.01^a^	2.00^a^	2.06^a^	0.03	0.008
N-balance (%)	73.95^d^	76.14^c^	76.63^b^	78.03^a^	0.22	< 0.001
Urinary-N: Fecal-N	0.33	0.29	0.27	0.26	0.03	0.133

^a^^, b, c, ..^ means in each row with different superscripts vary significantly (P < 0.05). CON: control diet, Z: control diet + 1% zeolite, OP: 19% orange pulp diet, OPZ: 19% orange pulp + 1% zeolite

### Blood biochemical parameters and antioxidative status

The present data in Table 6, reveals the non-significant effect of both zeolite addition and dried OP incorporation in the diet on plasma concentrations of total protein, albumin, globulin, AST, ALT, and total lipids. However, rabbits fed OP, Z, or OPZ diets recorded significantly (P < 0.05) lower concentrations of cholesterol, LDL, and triglycerides, and higher HDL compared with those fed control one. Although total antioxidant capacity (T-AOC) were not significantly affected by the experimental diet, malondialdehyde (MDA) significantly (P < 0.05) decreased with OP, Z, and OPZ groups compared with the CON one.

**Table 6:** Effect of dried orange pulp inclusion with or without zeolite on blood biochemical and antioxidant parameters

Item	Experimental groups				± SE	*p*-value
	CON	OP	Z	OPZ		
Plasma biochemical parameters						
Total protein (g/dl)	5.41	6.03	5.49	6.22	0.41	0.248
Albumin (g/dl)	3.25	3.26	3.16	3.10	0.09	0.885
Globulin (g/dl)	2.16	2.77	2.33	3.12	0.49	0.111
AST (U/l)	48.93	45.40	46.13	45.60	1.79	0.115
ALT (U/l)	29.67	27.33	28.50	27.11	1.29	0.178
Total lipids (mg/dl)	191.67	172.96	176.67	156.30	17.89	0.125
Cholesterol (mg/dl)	164.44^a^	146.94^c^	158.61^b^	143.89^c^	2.84	< 0.001
LDL (mg/dl)	119.35^a^	99.63^b^	97.99^b^	75.14^c^	9.04	< 0.001
HDL (mg/dl)	56.29^d^	62.61^c^	68.94^b^	78.83^a^	2.91	< 0.001
Triglycerides (mg/dl)	153.69^a^	135.10^c^	145.84^b^	132.97^c^	3.83	< 0.001
Antioxidants parameters						
T-AOC (mmol/l)	0.79	0.83	0.80	0.86	0.04	0.674
MDA (nmol/ml)	3.44^a^	2.67^b^	2.41^bc^	2.11^c^	0.26	< 0.001

^a^^, b, c, ..^ means in each row with different superscripts vary significantly (P < 0.05). CON: control diet, Z: control diet + 1% zeolite, OP: 19% orange pulp diet, OPZ: 19% orange pulp + 1% zeolite. AST: aspartate aminotransferase, ALT: alanine aminotransferase, LDL: low-density lipids, HDL: high-density lipids, T-AOC: total antioxidant capacity, MDA: malondialdehyde

### Cecum characteristics

The current data indicated significant (P < 0.05) decreases in cecum pH values (Table 7) when dried OP was included in diets either alone or in combination with zeolite being 6.08 and 6.29, respectively. However, no significant difference was detected between the Z and control groups. Values of cecum weight and length were not significantly affected by the treatment. Regarding the cecum microbiota, although total bacterial counts were not significantly altered, the experimental groups showed a significant (P < 0.05) decrease in the count of *E. coli* compared with the control.

**Table 7:** Effect of dried orange pulp inclusion with or without zeolite on cecum characteristics

Item	Experimental groups				± SE	*p*-value
	CON	OP	Z	OPZ		
Cecal pH	6.85^a^	6.08^b^	6.44^ab^	6.29^b^	0.61	0.021
Cecum weight (g)	47.67	45.67	48.33	46.33	1.59	0.261
Cecum length (cm)	53.33	51.33	53.67	51.67	0.17	0.329
Cecum microbiota						
Total bacteria (log CFU/g)	5.77	5.58	5.60	5.56	0.11	0.944
*E. coli* (log MPN/g)	2.72^a^	2.28^b^	2.27^b^	2.14^b^	0.30	0.008

^a^^, b^ means in each row with different superscripts vary significantly (P < 0.05). CON: control diet, Z: control diet + 1% zeolite, OP: 19% orange pulp diet, OPZ: 19% orange pulp + 1% zeolite

## Discussion

### Nutrients digestibility and nutritive value

In the current study, the impact of incorporating dried OP, natural zeolite, or both in diets of growing rabbits on digestibility, nutritive value, N-balance, blood metabolites, antioxidant status, cecum characteristics, and microbiota was evaluated. The improvement in nutrients digestibility in the current study as a result of dried OP inclusion in rabbitʼs diets were in agreement with Suliman et al. (2019) who recorded significant increases in digestibility of DM, OM, CP, and CF when dried OP was included at 8% of the rabbits’ diet while using OP at 16% had no significant effect on digestibility. Also, it was observed that replacing yellow corn in New Zealand rabbitʼs diets with 20, 40, or 60% dried OP, non-significantly improved the digestibility of DM and OM, and significantly the digestibility of CF (Ibrahim et al. 2011). In contrast, the inclusion of dried OP in rabbitʼs diets either at 5, 10, 15, 20 and 25% (Hon et al. 2009) or 1.5, 3 and 6% of the diet (Zeweil et al. 2015) had no significant effect on digestibility of DM, OM, and CP. These variations among the previous studies in nutrient digestibility as a result of dried OP incorporation may be attributed to the variability in the level of OP, diet composition, animal species, and the method of pulp processing (Bampidis and Robinson 2006).

The improvement in nutrient digestibility may be due to the high fiber content of dried OP which decreases the passage rate and increase the digesta retention time in the rabbitʼs gut which leads to more utilization of dietary nutrients (Fraga et al. 1991). Citrus (orange) pulp is characterized by its high content of soluble dietary fibers in the form of soluble non-starch polysaccharides (NSP), pectins, and gums (Mourão et al. 2008). Furthermore, Miron et al. (2002) stated that citrus pulp is considered a source of neutral detergent fiber (NDF) and soluble carbohydrates with less lignin content. In the present study, OP diets had more NDF, ADF, hemicellulose, and cellulose and less ADL compared with the CON diet, which reflects higher CF digestibility. Hon et al. (2009) showed that dried OP contains highly digestible fibers which are very important in rabbit nutrition in comparison to poultry (Zeweil et al. 2015). A negative correlation between dietary CF and utilization of other nutrients especially CP digestibility was reported by Adegbola and Okonkwo (2002). Therefore, the improvement in CF and other nutrient digestibility with OP diets in the current study, confirms the high contents of soluble carbohydrates and fibers in dried OP. The higher fiber digestibility with OP inclusion could be explained by high NFC (non-fiber carbohydrates) content that activates the polysaccharides degrading enzymes (Zhao et al. 2013). However, the values of EE digestibility among different experimental diets were not statistically significant in the current study, there were improvements with OP diets. The antioxidant content in citrus pulp may have a role in increasing EE digestibility (Lima et al. 2014).

Regarding the effect of zeolite addition alone (Z diet) on nutrients digestibility, the current findings are in parallel to the results obtained by Balakirev et al. (2000) who noted no significant effect of 1 and 5% zeolite in rabbitʼs diets on digestibility of OM, CP, and EE, while significant increases in digestibility were observed with 3% zeolite. However digestibility of DM, OM, and CP was not significantly different, the Z diet significantly increased the digestibility of CF. The positive effect of zeolite addition on digestibility especially fibers may be attributed to the high attraction between zeolite, active cations, and water which alters the passage rate of feed in the animal gut and improves fiber digestion (Johnson et al. 1988). Furthermore, the zeolite may have the ability to regulate the gut pH which promotes microbial fermentation and result in more fiber digestibility as a result of the buffering capacity of magnesium and aluminum silicate contents in a zeolite (Khachlouf et al. 2018), or due to its binding with H_2_ ions produced from the fermentation of dietary organic acids (Dschaak et al. 2010). Furthermore, it was indicated that zeolite administration can increase the hypertrophied function either of the intestinal villi or the epithelial cells in the ileum and duodenum, which can provide more surface area for nutrients absorption (Khambualai et al. 2009).

Regarding the nutritive value, the significant (P < 0.05) increases in TDN and DE values with OP, Z, and OPZ diets may be due to the improvement in nutrient digestibility with these diets compared with the control. The highest (P < 0.05) value of DCP was recorded for the OPZ diet, which may be attributed to the highest digestibility of CP compared with other diets. Consistent with the previous results, Ibrahim et al. (2011) recorded significant increases in TDN and DE values with non-significant differences in DCP values when dried OP was included in diets of rabbits at the level of 3.6, 7.2, and 10.8%. However, no significant effects of OP inclusion on nutritive value either as TDN, DE, or DCP were reported with rabbits (Hon et al. 2009; Zeweil et al. 2015; Suliman et al. 2019).

### Growth performance

It was observed in the current study, that the inclusion of dried OP in rabbitsʼ diet significantly (P < 0.05) improved ADG, which may be explained by the increase in nutrient digestibility with OP diets. Coincidence with that result, it was demonstrated that using orange peel extract in rabbitsʼ diet (Hassan et al. 2021) or using dried OP in broilersʼ diet (Abbasi et al. 2015) significantly increased ADG. It was reported by Hassan et al. (2021) that orange peel contains a significant amount of ascorbic acid (59 mg/100 g DM), which may enhance rabbit's growth performance, as it is considered a natural growth promoter due to its antioxidant effect that inhibits the cell damage (Qi et al. 2020), and prevents infection either by viruses or pathogenic bacteria (El-Desoukey et al. 2018). Furthermore, several active phenolic compounds were reported in orange peel such as flavonoids, flavones, isoflavones, coumarins, catechins, lignans, and β-carotene (Sir Elkhatim et al. 2018). These phenolic compounds were found to have a positive effect on digestive enzymes and saliva that may increase nutrient digestibility as shown in the current study, which in turn can improve animal growth (Hashemi and Davoodi 2010). Also, pectin content in the citrus peel can treat diarrhea as a result of its high-water absorbing property (Ibrahim et al. 2011), which may be reflected in higher growth performance. On the other hand, the incorporation of dried OP (Hon et al. 2009; Ojabo et al. 2012; Suliman et al. 2019) or citrus pulp (Lu et al. 2018) in diets growing rabbits, had no significant effect on ADG.

The present results showed also a significant (P < 0.05) increase in ADG with zeolite supplementation, which could be attributed to higher TDN and DE values for the Z diet compared with the control. In agreement with the previous result, Balakirev et al. (2000) recorded higher ADG for rabbits fed diets supplemented either by 1 or 3% zeolite. However, in other studies, ADG was not significantly changed by zeolite addition either in diets of rabbits (Fortomaris et al. 2007) or broilers (Schneider et al. 2016; Abdulrahman et al. 2022). The positive impact of zeolite supplementation on rabbits ADG may be attributed to its ability to capture harmful substances such as ammonia, mycotoxins, heavy metals, etc., and suppresses their negative effect on the health and performance of the animal (Stojković et al. 2012). Furthermore, Karamanlis et al. (2008) demonstrated that zeolite can increase the utilization of dietary nutrients and improve animal performance through the following 5 mechanisms: (1) binding with ammonia produced in the intestine and eliminates its toxic effect, (2) slower the digesta transit through the intestine, (3) reducing the absorption of some toxic compounds that produced from microbial degradation in the intestine as *p*-cresol, (4) enhancing the activity of pancreatic enzymes and (5) elimination the inhibitory effect of mycotoxin.

The non-significant differences recorded in the current study in DMI among the different experimental groups are in parallel with those obtained by Hon et al. (2009), Ojabo et al. (2012), Zeweil et al. (2015) and Lu et al. (2018) when either OP, orange peel or citrus pulp were incorporated in rabbitsʼ diet. In the same context, DMI was not significantly altered with zeolite addition to broilersʼ diets (Schneider et al. 2016). Contrary, significant increases in DMI were observed with orange pulp or peel inclusion in the diets of rabbits (Hassan et al. 2021) or broilers (Abbasi et al. 2015; Abdulameer 2018). Also, Fortomaris et al. (2007) reported significant increases in DMI when zeolite was added to rabbit diets at the level of 1.25 and 2.5%.

In concordance with the present data, non-significant differences were found in FCR values when rabbit's diet contained OP (Hon et al. 2009), orange peel extract (Hassan et al. 2021), or citrus pulp (Lu et al. 2018). While Suliman et al. (2019) recorded significantly higher values of FCR when 8 and 16% of dried OP were included in rabbitsʼ diet. Furthermore, no significant effect on FCR was conducted by Fortomaris et al. (2007) when zeolite was added at 1.25% of rabbit's diet. While Balakirev et al. (2000) mentioned that FCR was decreased with 1 and 3% zeolite addition to rabbit's diet.

### Nitrogen balance

In the present study, all the experimental groups showed similar values of N-intake, which may be due to that all diets were formulated to be iso-nitrogenous. Meanwhile, significant (P < 0.05) decreases in the values of total N-excreted and significant increases (P < 0.05) either in the values of N-retention or N-balance were observed with OP, Z, and OPZ groups. In matching with the previous findings, Ferrer et al. (2021) reported significant decreases in NH_3_ emission and NH_3_ content in Pigsʼ slurry when OP was included in the diets in two forms (dried and sun-dried silage). Similarly, Burmańczuk et al. (2015) found that adding zeolite at 5% of broilersʼ diet significantly decreased ammonia volatilization by about 33%. Also, N in pigs’ manure was reduced by 15 and 22% when zeolite was added to diets in 2 different particle sizes (Leung et al. 2007).

It was indicated that the inclusion of indigested dietary fiber ingredients in the form of non-starch polysaccharides (NSP) such as OP (Mourão et al. 2008; Hon et al. 2009), which includes hemicellulose, cellulose, fructans, galactomannans, glucomannans and pectins (Grieshop et al. 2001), is effective in altering the excretion pattern of nutrients and reducing the loss of odor compounds in feces (Mroz et al. 2000). Furthermore, Noblet and Goff (2001) illustrated another mechanism that may be involved in reducing NH3 emission from pigʼs slurry with OP diets, as it increases the microorganismsʼ fermentation of the total dietary fibers in the hindgut which leads to higher VFA production and decreases the slurry pH.

Ferrer et al. (2021) suggested that the decrease in NH3 emission with diets containing OP may be due to not only the decrease in total N excretion but also a result of N partition between urine and feces. Several studies confirmed that total dietary fibers; especially soluble fibers; may have the ability to alter N partitioning from urine to feces, which leads to increased N excretion in feces and a decrease in its content in urine as urea (Ferrer et al. 2018). Higher NDF intake with OP diets is another possible explanation for N partitioning (Ferrer et al. 2021). Similar findings were reported in N partitioning when different fibrous by-products were included in pigsʼ diets (Antezana et al. 2015). Furthermore, the obtained results in the current study, suggest that natural zeolite can be used as a feed additive in rabbitsʼ diets to reduce NH3 volatilization. That result is probably attributed to the ability of zeolite to bind ammonia that occurs in the digestive tract or due to its positive effect on CP digestibility (Karamanlis et al. 2008).

### Blood biochemical parameters and antioxidative status

Regarding blood biochemical, the current data indicate non-significant differences among the experimental groups in plasma total protein, albumin, globulin, AST and ALT activity, and total lipid concentrations, which are in harmony with the findings obtained by Lu et al. (2018) and Hassan et al. (2021) with rabbits when OP was included in diets. Moreover, the addition of zeolite in broilersʼ diet had no significant effect on blood total protein, albumin, globulin, AST, and ALT levels (Abdulrahman et al. 2022).

In agreement with the current results, significant decreases in concentrations of blood cholesterol, triglycerides (Abbasi et al. 2015), and LDL (Hassan et al. 2021) either with dried OP or OP extract inclusion in broiler or rabbitsʼ diets, in the same order. However, OP had no significant effect on cholesterol and triglyceride concentrations in other studies (Abdulameer 2018; Suliman et al. 2019). The ascorbic acid content in OP was found to have a role in preventing lipids peroxidation (Padayatty and Levine 2016). Also, its antioxidant effect which reduces cholesterol and triglycerides levels in blood and tissues may be attributed to its role in cholesterol hydroxylation in bile acid, because it acts as a cofactor of the 7-alpha-hydroxylase enzyme (Samman et al. 1997). Polidori et al. (2004) declared the inhibitory effect of ascorbic acid on LDL oxidation due to the reduction in levels of lipid peroxide in blood. Moreover, Njus et al. (2020) showed the ability of ascorbic acid to reduce the development of hypercholesterolemia in rabbits. Furthermore, it was indicated that the isoflavones content in orange peel could affect lipid metabolism as a result of reducing cortisol levels (El-Shazly et al. 2017), regulating cholesterol homeostasis (Medjakovic et al. 2010), upgrading LDL receptors (Fukuchi et al. 2008) and stimulating gene expression of essential protein and enzymes in lipid metabolism (Ezekwesili-Ofili and Gwacham 2015). Another explanation for reducing cholesterol and triglycerides with OP, is its pectin content which prevents bile acid reabsorption and consequently increases lipase activity (Bok et al. 1999). Moreover, it was evident that citrus flavonoids such as naringin and hesperidin play a role in the reduction of cholesterol and triglycerides due to the inhibition of hepatic enzymes (Lee et al. 2003) or regulation of glucose metabolism and lipogenesis (Evans et al. 2004). On the other hand, it was suggested that zeolite can reduce the total cholesterol level in the blood by adsorbing bile salts in the digestive tract, which subsequently increases the bile salts synthesis (Prvulović et al. 2007). Also, the hypocholesterolemic effect of the zeolite may be explained by the alteration in the rate of dietary cholesterol absorption (Alexopoulos et al. 2007) or due to short-chain fatty acid absorption by zeolite (Ly et al. 2007).

However, T-AOC did not significantly alter either with OP or zeolite diets, the antioxidant status of rabbits was improved as a result of significant (P < 0.05) reduction in MDA by 22.38, 29.94, and 38.66% in OP, Z, and OPZ groups, respectively. Similarly, Hassan et al. (2021) reported a non-significant effect of OP on T-AOC. On the other side, T-AOC was significantly increased with zeolite addition only when broilersʼ diet was contaminated with aflatoxin B1 (Abdulrahman et al. 2022). It was confirmed that citrus contains more than 60 flavonoids (Benavente-Garcia and Castillo 2008). The digestion of these compounds in the small intestine can result in increasing their level in the blood (Walsh et al. 2009). These flavonoids showed anti-inflammatory, antioxidant, and immune stimulation effects (Harborne and Williams 2000). A relationship between these phenolic compounds and T-AOC was demonstrated by Lu et al. (2018). Moreover, Anticona et al. (2020) reported high antioxidant activity in orange peel extract. The higher plasma T-AOC with dried OP diets may be ascribed to the content of phenolic compounds in OP that may inhibit the free radical activity and protect the animal tissues from lipid peroxidation (Zheng et al. 2009), or show a metal chelation activity (Andjelković et al. 2006).

Malondialdehyde (MDA) the oxidative stress marker, is produced as a result of lipids (polyunsaturated fatty acids) peroxidation (Kasperska-zajac et al. 2008). Qu et al. (2019) indicated the role of zeolite as an antioxidant feed additive in reduction of MDA concentration in broilers. The reduction effect on lipid peroxidation may be attributed to the zeolite properties as ion exchange, adhesion, adsorption, and cation binding such as binding with oxygen, the one of lipid peroxidation substrates (Pavelic et al. 2002). Consistently, Wu et al. (2013a) demonstrated a significant decrease in MDA with 2% zeolite supplementation in broilers' diets. Also, supplementation of turkey diets either with 1 or 2% zeolite significantly reduced MDA concentrations in both meat and liver (Hcini et al. 2018). The same authors explained the detoxification effect of zeolite which decreases lipid peroxidation as a result of free radical scavenging. Pavelić et al. (2018) refered to the role of zeolite as an indicator of improving animal immunity. Several mechanisms were reported to explain that positive effect, either by reducing the possibility of some metabolic diseases in animals such as diarrhea and hypocalcemia (Stojković et al. 2005) or due to the detoxification property of zeolite as a result of its ability to bind several harmful substances as mycotoxins, radioactive elements and poisons (Katsoulos et al. 2015). Also, the improvement in blood globulin levels by incorporating OP in the diets may be attributed to the steroidal flavonoids content in OP that stimulates cortisone secretion, as 80–90% of cortisone was found to bind with globulin (Gardill et al. 2012).

### Cecum characteristics

In accordance with the current results, Ferrer et al. (2021) reported a reduction in the pH of pigsʼ slurry when OP was included in diets either dehydrated or sun-dried silage. Higher fermentation of dietary fibers with OP incorporation, which subsequently increases VFA production (Noblet and Goff 2001), maybe the reason for lower pH values. Also, Wu et al. (2013b) reported lower cecal pH values with synthetic zeolite, while no significant difference was observed in pH value between the natural zeolite-supplemented group and control. The high affinity of zeolite for active cations and water was suggested to improve osmotic activity and fermentation process in the gut, which may regulate pH by acting as a buffer for hydrogen ions produced from organic acids fermentation (Dschaak et al. 2010). Furthermore, a relationship between the viscosity of ingesta, its density, and pH value was observed. As viscosity increases, as a result of zeolite administration, the ingesta density and intestinal pH decrease (Ismail et al. 2011).

Consistent with the current data of cecum microbiota, Alefzadeh et al. (2016) recorded significant decreases in *E. coli* with 2 or 4% orange peel powder included in broilersʼ diets. In the same context, Abbasi et al. (2015) reported significant decreases in cecal *E. coli* when broilersʼ diets contained 0.5% dried OP. However, the same authors showed that using dried OP at 1, 1.5, or 2% of the diet had no significant effect on *E. coli* count. That reduction effect of OP could be related to the inhibitory effect of OP essential oils on *E. coli* count (Lee et al. 2014). Moreover, the saponins, tannins, essential oils, phenolic compounds, and flavonoids contents in both citrus pulp and peel were found to have antibacterial activity (El-Desoukey et al. 2018), which can inhibit the synthesis of cell protein (Shetty et al. 2016). Consistently, zeolite addition exhibited a reduction in *E. coli* counts either in the pigsʼ diet (Wang et al. 2021) or in the broilersʼ diet (Wu et al. 2013b). The lower count of *E. coli* with zeolite may be a result of good microbial environmental balance, or due to the catalytic property of zeolite that can enhance the activity of enzymes, alter the pH of the digestive tract, and improve lumen ionic composition (Wang et al. 2021).

## Conclusion

Therefore, the current data indicated that a combination of dried orange pulp and natural zeolite (OPZ) in diets of growing rabbits can improve nutrients digestibility, the measurements of feed energy and protein values, average daily gain, feed conversion ratio, N-retention and antioxidative status of the animals. Furthermore, the OPZ diet exhibited the lowest concentrations of plasma cholesterol, LDL, triglycerides, and cecal count of *E. coli*.

## Data Availability

The data presented in this study are available upon request to the corresponding author.
